# CARING FOR OLDER INDIGENOUS PEOPLE WITH CO-MORBIDITIES AT END OF LIFE

**DOI:** 10.1093/geroni/igz038.2202

**Published:** 2019-11-08

**Authors:** Kathleen R Mason, Tess H Moeke-Maxwell, Merryn Gott

**Affiliations:** University of Auckland, Auckland, New Zealand

## Abstract

The number of deaths among older Māori, the indigenous people of New Zealand, are expected to increase by 48% by 2030. Colonization has had a varied impact on Māori ways of being and end-of-life care has become more difficult. Many have become disenfranchised from their families, peoples, lands and culture. Pae Herenga, a for-Māori by-Māori with-Māori qualitative research project, investigated the traditional Māori end-of-life care customs that Māori families used while caring for someone who was dying. An online education resource was developed to support Māori families, their communities and the palliative care sector. Interviews were conducted with 60 Māori participants including older many people (aged over 70). The findings found that families rich in cultural knowledge were proficient in caring for a loved one at end-of-life irrespective of their social or economic position. Cultural care values such as unconditional love, companionship, reciprocity, supportive relationships and collective decision making safeguarded care preferences of the dying. Access to traditional knowledge and traditional healing practices, and an understanding of spirituality helped to strengthen and prepare the dying person, and their families, on the end-of-life journey. The study also found that those families connected to communities’ rich in Māori cultural resources, such as knowledgeable older Māori people, were well supported by the community at end-of-life. This study highlights that Māori use of traditional care customs in all care settings can better support a ‘good death’ from a cultural perspective.

